# Sumo-dependent substrate targeting of the SUMO protease Ulp1

**DOI:** 10.1186/1741-7007-9-74

**Published:** 2011-10-28

**Authors:** Zachary C Elmore, Megan Donaher, Brooke C Matson, Helen Murphy, Jason W Westerbeck, Oliver Kerscher

**Affiliations:** 1 Biology Department, The College of William & Mary, ISC3047, 540 Landrum Drive, Williamsburg, VA 23185, USA

## Abstract

**Background:**

In the yeast *Saccharomyces cerevisiae*, the essential small ubiquitin-like modifier (SUMO) protease Ulp1 is responsible for both removing SUMO/Smt3 from specific target proteins and for processing precursor SUMO into its conjugation-competent form. Ulp1 localizes predominantly to nuclear pore complexes but has also been shown to deconjugate sumoylated septins at the bud-neck of dividing cells. How Ulp1 is directed to bud-neck localized septins and other cytoplasmic deconjugation targets is not well understood.

**Results:**

Using a structure/function approach, we set out to elucidate features of Ulp1 that are required for substrate targeting. To aid our studies, we took advantage of a catalytically inactive mutant of Ulp1 that is greatly enriched at the septin ring of dividing yeast cells. We found that the localization of Ulp1 to the septins requires both SUMO and specific structural features of Ulp1's catalytic domain. Our analysis identified a 218-amino acid, substrate-trapping mutant of the catalytic domain of Ulp1, Ulp1(3)^(C580S)^, that is necessary and sufficient for septin localization. We also used the targeting and SUMO-binding properties of Ulp1(3)^(C580S) ^to purify Smt3-modified proteins from cell extracts.

**Conclusions:**

Our study provides novel insights into how the Ulp1 SUMO protease is actively targeted to its substrates *in vivo *and *in vitro*. Furthermore, we found that a substrate-trapping Ulp1(3)^(C580S) ^interacts robustly with human SUMO1, SUMO2 and SUMO2 chains, making it a potentially useful tool for the analysis and purification of SUMO-modified proteins.

## Background

Cell division is a fundamental feature of all life and involves the controlled duplication and faithful segregation of an organism's genetic material from one cell to the next. In eukaryotes, each cell division cycle is therefore executed as a tightly regulated, stepwise program that relies on intact chromosomes. In humans, the consequences of faulty chromosome segregation and the inability to repair DNA damage have been implicated in cancer, aging and congenital birth defects.

Ubiquitin and small ubiquitin-like modifier (SUMO), two small proteins that can become attached to other cellular proteins in a reversible manner [1], control important aspects of the cell division program. Ubiquitin is best known for its role in the targeted, proteasome-mediated destruction of proteins, including key cell-cycle regulators, but also holds nonproteolytic functions [2]. Sumoylation, on the other hand, does not directly target proteins for degradation. Rather, modification of proteins with SUMO has been shown to modulate various cellular processes, including cell-cycle regulation, transcriptional activation, nucleocytoplasmic transport, DNA replication and repair, chromosome dynamics, apoptosis, ribosome biogenesis, and the formation of nuclear bodies [3]. Additionally, an unexpected role of SUMO in protein ubiquitination has been uncovered. Briefly, degradation of several nuclear proteins, including some that are involved in DNA repair and transcriptional regulation, are preceded by modification with SUMO. These sumoylated proteins are recognized by SUMO-targeted ubiquitin ligases (STUbLs), which mediate their ubiquitination [4].

SUMO proteins are highly conserved from yeast to humans. Yeast cells express one SUMO protein (Smt3), and vertebrates express three isoforms (SUMO1, SUMO2 and SUMO3) [5]. SUMO2, SUMO3 and yeast Smt3 can form SUMO chains. SUMO1, on the other hand, lacks the internal lysine required for polymerization and may function as a chain terminator for SUMO2 and SUMO3 chains [6]. All SUMO variants are conjugated to lysine residues of specific proteins, but only a fraction of these target proteins are modified with SUMO at any given time [7,8]. In metazoans, the dysregulation of sumoylation adversely affects developmental processes and has been implicated in the progression of neurodegeneration, cancer and infectious diseases [9,10]. More than 1, 000 sumoylated proteins have been identified in yeast and humans, but only in a few cases has the role of sumoylation been studied in detail [11].

In the budding yeast *Saccharomyces cerevisiae*, the ligation of SUMO to specific substrate proteins requires an E1 heterodimer (Aos1 and Uba2) that activates SUMO, as well as E2 (Ubc9) and E3 (Siz1, Siz2, and Mms21) enzymes that aid in the conjugation and ligation of SUMO to proper target proteins [1]. Two yeast SUMO proteases, Ulp1 and Ulp2, contain a conserved cysteine protease domain that can remove the SUMO moiety from modified proteins. Recent evidence suggests that Ulp2, and its mammalian orthologs Susp1/SENP6 and SENP7, play a role in the removal of SUMO and SUMO chains from nuclear proteins [12-16]. Ulp1, on the other hand, has two contrasting cellular functions. Ulp1 facilitates sumoylation by processing precursor SUMO into its conjugation competent form. Conversely, Ulp1 also facilitates desumoylation by removing SUMO from nuclear and cytosolic proteins after conjugation [17]. Therefore, impairment of Ulp1 results in the accumulation of SUMO conjugates and the inability to carry out *de novo *sumoylation. The resulting lack of mature SUMO has been shown to adversely affect cellular DNA repair processes, the processing and export of the 60S preribosomal particle, nucleus-cytoplasm trafficking and cell viability [18-21].

The substrate specificity of SUMO proteases is at least in part regulated through their localization [22]. For example, certain yeast (Ulp2) and vertebrate (SENP6 and SENP7) SUMO proteases localize within the nucleus. In contrast, both yeast (Ulp1) and vertebrate (SENP1 and SENP2) SUMO proteases reside at the nuclear envelope (NE) through their interactions with the nuclear pore complex (NPC) [23-26]. Distinct domains have been identified that are required for Ulp1 NPC localization (amino acid residues 1 to 403) and SUMO processing (amino acid residues 404 to 620) [25-28]. The Ulp1 localization domain promotes interaction with karyopherins, which are soluble proteins that mediate transport across the NE and help localize Ulp1 to the nucleoplasmic side of the NPC. The Ulp1 localization domain can be subdivided into region 1 (Kap121-binding domain) and region 2 (Kap60- and Kap95-binding domain). Juxtaposed to the NPC localization domain of Ulp1 is a coiled-coil (cc) domain with a putative nuclear export signal and region 3, the catalytically active, conserved ubiquitin-like protease domain (UD) of Ulp1 [25-27]. Only regions 1 and 2 are involved in Ulp1 localization to the NPC, and the karyopherins seem to play a redundant role. NPC association of Ulp1 requires several proteins, including the nucleoporins Nup60 and Nup84, the silencing protein Esc1 and the myosin-like proteins Mlp1/2 [18,19,28]. Together these proteins may provide a scaffold for the functional regulation and substrate access of Ulp1 at the NPC.

The identification of NPC localization domains in Ulp1 has done little to aid our understanding of how SUMO proteases are targeted to their respective substrates [26]. One possibility is that SUMO proteases may contain structural features which allow for noncovalent interactions with SUMO and SUMO-modified proteins as they enter the nucleus. Indeed, conserved SUMO-interacting motifs (SIMs) have been predicted to be localized in the yeast SUMO protease Ulp2, as well as in mammalian SENP1, SENP2, SENP6 and SENP7 [14,22,29,30]. Even though SIMs have not been identified in Ulp1, the crystal structure of the catalytic domain (region 3) bound to Smt3 reveals that both proteins interact through multiple residues that are distributed across a SUMO-binding surface (SBS) on the SUMO protease [31]. Only the carboxy terminus of bound Smt3 is inserted into a hydrophobic tunnel that leads toward Ulp1's active site. SUMO processing and deconjugation require an active site cysteine residue that resides at the end of this tunnel (see Additional files 1 and 2). It has been suggested that this configuration may allow for the accommodation of many different sumoylated proteins as well as SUMO precursors [31].

Ulp1 and several other SUMO proteases play important roles in mitosis [17,32]. In budding yeast, loss of Ulp1-mediated desumoylation leads to cell-cycle progression defects and cell death [17]. This observation suggests that Ulp1 plays a key role in the sumoylation dynamics of important cell-cycle regulatory proteins. Though these cell-cycle-specific targets have eluded identification, several nuclear and cytosolic proteins involved in DNA replication and mitosis have been identified as Ulp1 desumoylation substrates [33-35]. How the NPC-localized Ulp1 is targeted to these mitotic substrates, especially those that are localized in the cytosol, is not entirely clear. In budding yeast, the NE does not break down during mitosis, and access to cytosolic desumoylation targets is therefore not automatic. It has been reported that during mitosis, Kap121 blocks Ulp1's access to its NPC-binding site and thus promotes an interaction of Ulp1 with septins [27]. A deletion mutant of Ulp1 lacking region 2 (Δ2), the Kap60- and Kap95-binding domain, has previously been shown to localize to septins in a Kap121-dependent manner [27]. Curiously, it has recently been demonstrated that region 2 also plays a role in nucleolar accumulation of Ulp1 after ethanol-induced stress [36].

One set of cytosolic substrates of the Ulp1 SUMO protease are the septins [27,35]. The septins comprise an evolutionarily conserved class of GTPases that are implicated in bud-site selection, bud emergence and growth, microtubule capture and spindle positioning [37]. Members of the septin family in yeast include Cdc3, Cdc10, Cdc11, Cdc12 and Shs1/Sep7. These proteins are unique because they can form filaments that assemble into a ring structure and mark the site of new bud formation during cell division. At the end of mitosis, this ring separates and resembles a double-collar residing at the junction between the mother and daughter cells.

The septins Cdc3, Cdc11 and Shs1 are subject to sumoylation. Sumoylation of the septins occurs very briefly from the onset of anaphase to cytokinesis, with SUMO being attached only to the mother side of the double-septin ring collar [27,38,39]. Cell-cycle (G_2_/M) arrest with nocodazole, a microtubule depolymerizing drug, greatly increases SUMO conjugation to septins [38]. Septin sumoylation in budding yeast is mediated by the SUMO E3 ligase Siz1 [40,41]. During most of the cell cycle, Siz1 resides in the nucleus. However, at the M phase, Siz1 exits the nucleus to sumoylate septin proteins and possibly other cytosolic substrates [42]. Deletion of *SIZ1 *from cells abolishes septin sumoylation while causing only mild growth and cell-cycle progression defects. At the end of mitosis, the septins are desumoylated by Ulp1, even though Ulp1 remains visibly enriched at the NPC [27,35,38].

In the current study, we focused on the SUMO protease Ulp1. As detailed above, Ulp1 resides at the inner face of the NPC. This enrichment at the NPC depends on direct interactions with karyopherins and two domains in the amino terminus of Ulp1. In theory, this localization is well-suited to give Ulp1 access to some nuclear substrates and those in transit across the NE. However, both nuclear and cytosolic desumoylation targets of Ulp1 have been identified, posing an interesting question. How is Ulp1 directed to its cytosolic desumoylation targets? To answer these questions, we sought to identify features of Ulp1 required for substrate targeting *in vivo *and *in vitro*. Herein we describe our finding that the carboxy terminus of Ulp1 affects the targeting and retention of this SUMO protease to sumoylated target proteins, including septins, at the bud-neck of dividing cells. Specifically, we show that the interaction with SUMO comprises an important aspect of the subcellular targeting of Ulp1 to these substrates. Our findings are confirmed by biochemical analyses that focus on the SUMO-binding properties of Ulp1(3)^C580S^, a novel truncation mutant that interacts avidly with SUMO and sumoylated proteins *in vivo *and *in vitro*. Significantly, the results of this study add important new details to our understanding of how Ulp1 interacts dynamically with its substrates and also provides potentially useful new directions for the study of Ulp1-interacting proteins.

## Results

### Ulp1 localization to the nuclear envelope and the septin ring

**Figure 1:**
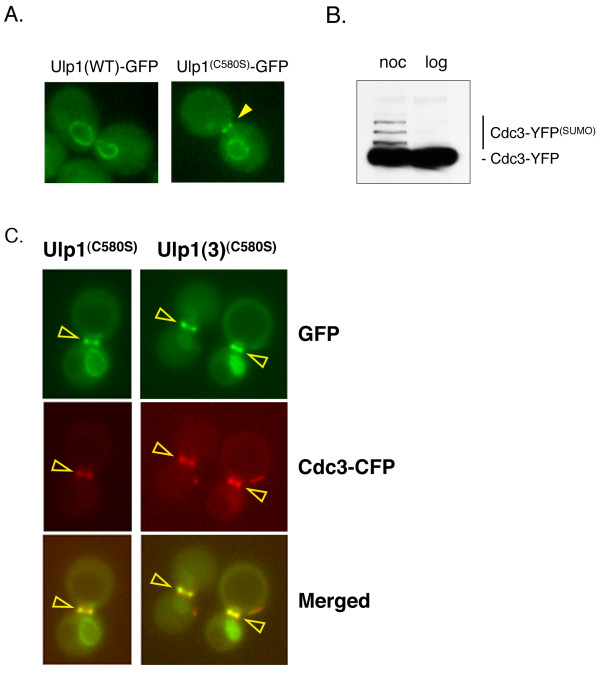
**Localization of Ulp1 and the catalytically inactive Ulp1^(C580S) ^in dividing yeast cells**. **(A) **Yeast cells (MHY500) were transformed either with a low-copy plasmid expressing GFP fusions of Ulp1 or with the catalytically inactive Ulp1^(C580S) ^mutant. Representative images indicating the localization of GFP-tagged Ulp1 and Ulp1^(C580S) ^after nocodazole-induced G_2_/M arrest are shown (YOK 1611 and YOK 1474). Note that only the Ulp1^(C580S) ^mutant can be seen at the bud-neck of arrested cells. The arrowhead indicates the position of the bud-neck. **(B) **Confirmation of sumoylation of Cdc3 was achieved. Whole-cell extracts (WCE) from yeast cells expressing the YFP-tagged septin Cdc3 (YOK 1398) were treated with nocodazole (noc) or grown logarithmically (log) prior to preparation of WCEs. Extracted proteins were then separated on SDS-PAGE gels and probed with the JL-8 antibody (see Methods) to detect Cdc3-YFP and more slowly migrating sumoylated Cdc3-YFP adducts. The identity of sumoylated Cdc3-YFP bands was confirmed by comparing gel shift assays with untagged and FLAG-tagged Smt3 (data not shown). **(C) **Colocalization of Cdc3 and Ulp1 is shown. A strain coexpressing full-length Ulp1^(C580S)^-GFP (green) and Cdc3-CFP (red) (strain YOK 2204) was arrested in G_2_/M and then observed under a fluorescence microscope with the appropriate filter sets (left panel). Arrowheads indicate septin-localized, pseudocolored Ulp1-GFP (green), Cdc3-CFP (red) and the merged image (overlay). Also shown for comparison (right panel) is the colocalization of the Ulp1(3)^(C580S)^-GFP truncation and Cdc3-CFP (strain YOK 2205). Ulp1(3)^(C580S)^-GFP is described in Figure 4.

As part of a larger study to identify how Ulp1 is targeted to its mitotic desumoylation substrates, we analyzed the localization of GFP-tagged versions of both the full-length wild-type (WT) Ulp1 and a catalytically inactive mutant of Ulp1 (Ulp1^C580S^) in G_2_/M-arrested yeast cells (see Methods and Additional file 3). The C580S mutation replaces the catalytic cysteine with a serine residue, rendering the Ulp1 SUMO protease catalytically inactive [17]. Both fusion proteins were expressed under the control of the Ulp1 promoter on low-copy plasmids, and images were collected using a fluorescence microscope. Consistent with its localization to NPCs, WT Ulp1 stained only the NE of arrested yeast cells (Figure 1A, left). Unexpectedly, however, full-length Ulp1^C580S ^was enriched at both the bud-neck and the NE of G_2_/M-arrested cells (Figure 1A, right). This bud-neck localization of Ulp1^C580S ^is reminiscent of the localization of the septin ring. Several sumoylated septins have been shown to be Ulp1 substrates, and we show in this study that the septin Cdc3 is highly sumoylated during G_2_/M arrest (Figure 1B). Furthermore, a catalytically inactive Ulp1 mutant colocalizes with the septin Cdc11 in G_2_/M-arrested (noc) cells (Figure 1C). Therefore, Ulp1^C580S ^resides at the bud-neck localized septin ring.

Our data suggest that introducing the C580S mutation into the catalytic domain of Ulp1 somehow alters the subcellular distribution of this SUMO protease, causing it to localize with a bud-neck-associated substrate, possibly a sumoylated septin protein. Localization changes have also been reported for catalytically inactive, substrate-trapping mutants of phosphatases that form stable complexes with their substrates *in vivo *[43].

### SUMO conjugation is required for Ulp1 localization to the septin ring

**Figure 2:**
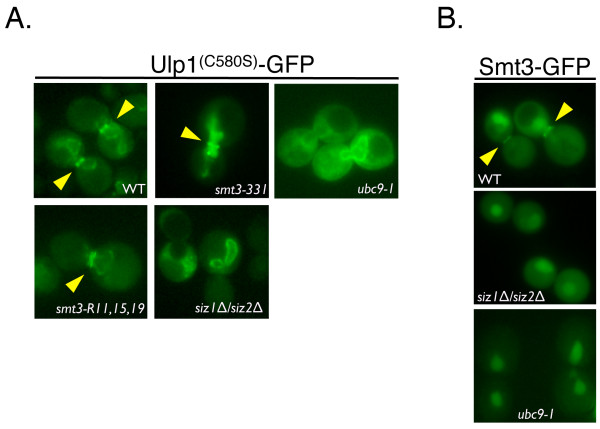
**Small ubiquitin-like modifier (SUMO) is required for the localization of Ulp1^(C580S) ^to the septin ring**. **(A) **The indicated mutants *smt3-331, ubc9-1, smt3-R11, 15, 19, siz1Δ siz2Δ *(YOK 1995, YOK 2065, YOK 1910 and YOK 2067) and a WT control strain (WT) were transformed with a plasmid expressing GFP-tagged Ulp1^(C580S)^. Representative images indicating the localization of GFP-tagged Ulp1^(C580S) ^after G_2_/M arrest are shown. The septin ring localization of Ulp1^(C580S) ^is indicated where present (arrowheads). Note that Ulp1^(C580S) ^failed to localize to the septin ring in SUMO-conjugating and ligating enzyme mutants (*ubc9-1 *and *siz1Δ siz2Δ*, respectively). **(B) **Septin ring localization of Smt3-GFP is absent in *ubc9-1 *and *siz1Δ siz2Δ *strains. Localization of Smt3-GFP was visualized in G_2_/M-arrested WT, *ubc9-1 *and *siz1Δ siz2Δ *strains (YOK 1857, YOK 2144 and YOK 2143) using fluorescence microscopy. Arrowheads indicate the position of the septin ring.

We tested whether the C580S mutation that visually increased the ability of Ulp1 to associate with the septin ring *in vivo *was, in fact, SUMO-dependent. For this purpose, the Ulp1^C580S ^construct was expressed in two Smt3 mutants (*smt3-331 *and *smt3-R11, 15, 19*) or two SUMO pathway mutants (*ubc9-1, siz1Δ siz2Δ*) [13,39,41,44-46]. Logarithmically growing cells of each mutant were arrested in G_2_/M, and images were collected to assess the septin ring localization of Ulp1^C580S ^in comparison to an *SMT3 *WT strain. In our analyses, we found that in the absence of both SUMO chains (in the R11, 15, 19 mutants) and a mutant SUMO protein (in the *smt3-331 *mutant), the localization of Ulp1^C580S ^to the septin ring was reduced (22% in *smt3-331 *and 36% in *smt3-R11, 15, 19*) in frequency and intensity but not abolished (Figure 2). We obtained different results in the *ubc9-1 *strain, a mutant of the SUMO E2-conjugating enzyme which impairs SUMO conjugation, and in the *siz1Δ siz2Δ *strain, a SUMO E3 ligase double-mutant that lacks sumoylation of septins and many other proteins [40,41,44]. Consistent with a role for Smt3 in the localization of Ulp1^C580S^, we were unable to detect septin ring localization of Ulp1^C580S ^in *ubc9-1 *and *siz1Δ siz2Δ *strains. However, Ulp1^C580S ^was retained at the NE (Figure 2A). As an additional control, the septin ring localization of GFP-tagged Smt3 was undetectable in both *ubc9-1 *and *siz1Δ siz2Δ *strains (Figure 2B).

Smt3 conjugation is required for Ulp1 localization to the septin ring. Therefore, Ulp1 is targeted to the septin ring of dividing cells in a SUMO-dependent fashion. Our data also suggest that the formation of SUMO chains on substrates may enhance this targeting of Ulp1.

### Distinct and separate Ulp1 domains are required for localization to the septin ring

**Figure 3:**
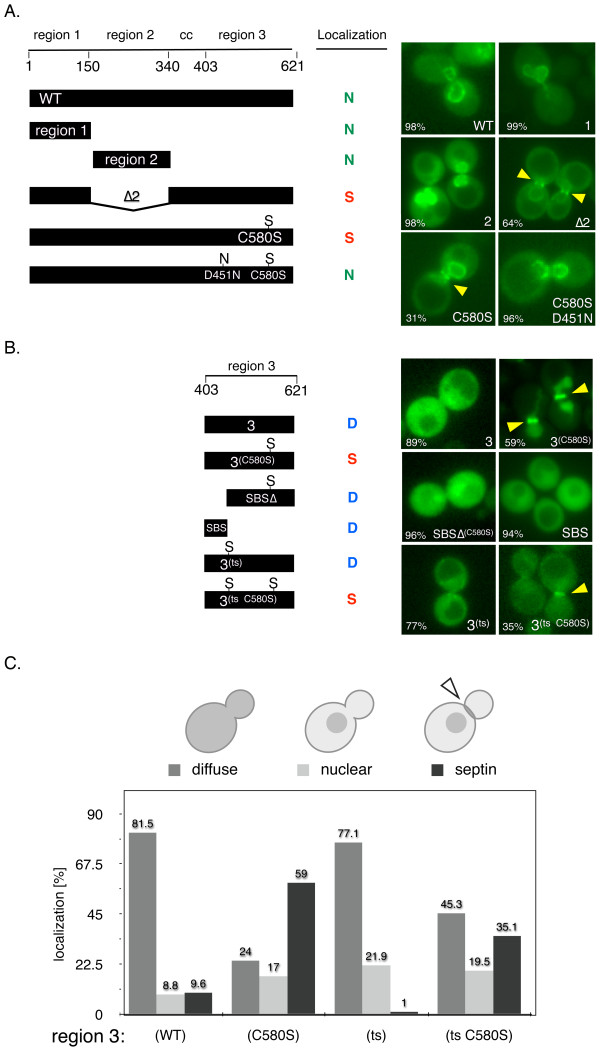
**Distinct and separate Ulp1 domains are required for localization to the septin ring**. **(A) **and **(B) **Left: A schematic of Ulp1 deletion and truncation mutants used in this study is shown. The length of each construct (amino acid scale 1 to 621), the individual domains of Ulp1 and pertinent amino acid changes are shown. WT: full-length Ulp1; region 1: Ulp1(1 to 150); region 2: Ulp1(151 to 340); region 3: Ulp1(341 to 621); Δ2: Ulp1 lacking region 2; C580S: catalytically inactivating mutation; D451N: deleted salt bridge with small ubiquitin-like modifier (SUMO) (YOK 1611, YOK 1474, YOK 1490, YOK 1861, YOK 1479, YOK 2016, YOK 1839, YOK 1907, YOK 1903, YOK 2203, YOK 1828 and YOK 2157). Colored letters N, S and D summarize the observed nuclear, septin and diffuse localization of the indicated constructs, respectively. SBS corresponds to a shallow SUMO-binding surface on Ulp1 [31,57,58]. Right: Representative images of G_2_/M-arrested cells expressing the GFP-tagged Ulp1 constructs shown on the left. The arrowheads indicate the fraction of cells (%) with N, S or D localization and the presence and position of septin ring-localized Ulp1 constructs. **(C) **Quantification of distinct subcellular localization of wild-type and mutant Ulp1 region 3 constructs. Large-budded G_2_/M-arrested cells were imaged to assess diffuse, nuclear or septin ring localization (*n *> 100).

Our finding that a single point mutation in Ulp1, C580S, dramatically enhanced the localization of full-length Ulp1 to the septin ring in a SUMO-dependent fashion warranted a more detailed analysis of the targeting domains in Ulp1. Therefore, we generated a collection of GFP-tagged Ulp1 truncations and domains that were expressed under control of the Ulp1 promoter. We reasoned that the truncations and domains of Ulp1 that retained substrate targeting information would also localize to the septin ring in G_2_/M-arrested cells. In all, we assessed the localization of ten GFP-tagged constructs in comparison to full-length WT Ulp1 and full-length Ulp1^C580S ^(C580S). Our choice of individual constructs was guided by previous findings that Ulp1 consists of functionally separate domains. These domains include a Kap121-binding domain with a role in septin localization (region 1), a Kap95- and Kap60-binding domain with a role in NPC anchoring (region 2), a cc domain harboring a nuclear export signal (CC) and the catalytic UD (region 3) [25-27]. Representative images of these domains and their subcellular localization are shown in Figures 3A and 3B. As previously reported, we found that the Ulp1 protein lacking region 2 (Δ2) localized to the septin ring in the majority of large-budded, arrested cells [27]. Therefore, region 2 of Ulp1 normally antagonizes localization and/or retention at the septin ring. This result is complemented by our novel finding that the full-length Ulp1^C580S ^localized to the septin ring in 33% of all arrested, large-budded cells (Figures 1A and 3A).

Next we investigated other residues of Ulp1 that could affect the septin ring localization of the Ulp1^C580S ^mutant, possibly by interfering with its targeting to sumoylated substrates. Aspartate 451 (D451) in Ulp1 is required to form an essential salt bridge with arginine 64 of Smt3 [31,47]. Therefore, we introduced a D451N mutation into Ulp1^C580S ^and found that it abolished the accumulation of the full-length Ulp1 double-mutant (D451N and C580S) at the septin ring (Figure 3A). This finding underscores the importance of Smt3 in targeting full-length Ulp1 to the septin ring shown in Figure 2. Additionally, it may indicate that D451 is required for targeting of sumoylated proteins and the C580S mutation is required for retention of Ulp1 at the septin ring.

Most intriguingly, we found that a truncation consisting only of region 3 with the C580S mutation (Ulp1(3)^(C580S)^) displayed robust septin ring localization in 59% of cells (Figures 1C, right panel, and Figure 3B). In stark contrast, regions 1 and 2 and WT region 3, lacking the C580S mutation, failed to localize to the septin ring (Figures 3A and 3B). However, in strains with diffuse Ulp1 truncations, the septin ring stays intact. Therefore, necessary and sufficient SUMO-dependent targeting information is contained in region 3 of Ulp1, but not in regions 1 and 2. The latter conclusion is confirmed by two-hybrid assays with Smt3.

The previously published cocrystal structure of Ulp1 with Smt3 (MMDB database 13315) reveals that amino acids 418 to 447 of region 3 make extensive contact with Smt3 and constitute an exposed SBS [31] (see also Additional files 1 and 2). The SBS is situated next to, but does not include, the critical D451 residue that contacts Smt3 [31,47]. Additionally, deletion of this SBS in region 3 of Ulp1 abolishes the complementation of a *ulp1Δ *deletion mutant [26]. In an attempt to identify critical residues in the evolutionary conserved SBS domain, we used psi-blast to compare the protein sequence of the yeast Ulp1 catalytic domain with all nonredundant protein sequences in the National Center for Biotechnology Information database for seven iterations and limited the output to the top 250 matches. Our results contained 81 different species. Among these species, 61% of the sequences were identified as verified or predicted sentrin/SUMO protease/Ulp1 genes, 24% were identified as unnamed protein products or hypothetical genes and 15% were classified as "other" (crystal structures, unanalyzed sequences and so on). The alignment of these sequences allowed us to identify areas of strong conservation (see Additional file 1). Using this approach, we identified several highly conserved residues in the SBS. However, these amino acids did not contact Smt3 in the published cocrystal structure and likely play structural roles in Ulp1 folding [31].

We investigated the effect of deleting the entire SBS domain on the localization of Ulp1(3)^(C580S)^. A Ulp1(3)^(C580S)^SBSΔ construct did not localize to the septin ring in the majority of cells (96%). These results match those obtained by Li and Hochstrasser [26] using a WT Ulp1(3)ΔSBS construct (C173). We confirmed that SBSΔ and other Ulp1(3) constructs are expressed as soluble proteins, suggesting that they are not grossly misfolded. We also cloned and expressed the SBS domain as a fusion with GFP (SBS-GFP). This construct was distributed diffusely throughout the cell and failed to localize to the septin ring (Figure 3, middle). These data suggest that the SBS domain of region 3 may be required for the initial interaction with sumoylated substrates, but additional features of Ulp1 are required for targeting (D451) and retention (C580S) of this SUMO protease at the septin ring.

Next we directed our attention to the temperature-sensitive *ulp1ts-333 *allele. This mutant allele causes cells to arrest in mitosis and accumulates unprocessed SUMO precursor and sumoylated proteins [17]. Our *ulp1ts *construct of region 3, Ulp1(3)^ts^, contains three mutations (I435V, N450S and I504T), and introduction of C580S into Ulp1(3)^ts ^showed a greatly reduced incidence and intensity of septin ring localization (compare panels in Figures 3B and 3C). We noted that the (N450S) mutation in the ts construct was located next to the salt bridge-forming residue D451 described above and that both residues were highly conserved in the consensus sequence of Ulp1-like molecules (Additional file 1). This suggests that residues altered in *ulp1ts-333*, specifically N450, may contribute to Smt3 interaction and possibly substrate targeting. It is possible that N450S perturbs the salt-bridge interaction formed between D451 of Ulp1 and R64 of Smt3, thus reducing the interaction with Smt3 and contributing to the temperature-sensitive phenotype. In support of this hypothesis, correction of the N450S mutation in Ulp1(3)^ts (S450N) ^partially rescued the slow growth defect of a *ulp1Δ *strain at 30°C and 37°C (data not shown). The effect of the *ulp1ts *mutation on Ulp1's ability to interact with Smt3 is explored in more detail below.

We tested which domains of Ulp1 are required for targeting and retention at the septin ring *in vivo*. Using our region 1 and region 2 GFP-tagged constructs (see Figure 3A), we show that septin-targeting information is not contained in the domains that are known to interact with karyopherins. The Δ2 construct recapitulates the previous finding that Kap121-binding to region 1 regulates access of Ulp1 to the septin ring. The full-length Ulp1^C580S ^mutant reveals that a single substrate-trapping mutation in Ulp1 suffices to enrich Ulp1 at the septin ring. To show that an Smt3 interaction is required for the septin localization of Ulp1^C580S^, we created the double-mutant (D451N and C580S). The D451N mutation is known to destroy an essential salt bridge formed between Smt3 and Ulp1. Next, using the Ulp1(3)^C580S ^construct, we show that the septin-targeting information is limited to region 3 of Ulp1 (Figure 3B). Further truncating Ulp1(3)^C580S ^revealed that a previously identified SBS domain in Ulp1(3)^C580S ^is also involved in septin targeting and retention. To test whether mutations found in region 3 of the *ulp1ts *mutant play a role in septin localization, we introduced three additional mutations, I435V, N450S and I504T, into Ulp1(3)^C580S^. This Ulp1(3)^ts C580S ^construct showed a reduced ability to enrich at the septin ring (Figure 3C), suggesting that its ability to interact with sumoylated septins may be reduced but not abolished.

### Kap121-independent SUMO-targeting information resides in the catalytic domain of Ulp1

**Figure 4:**
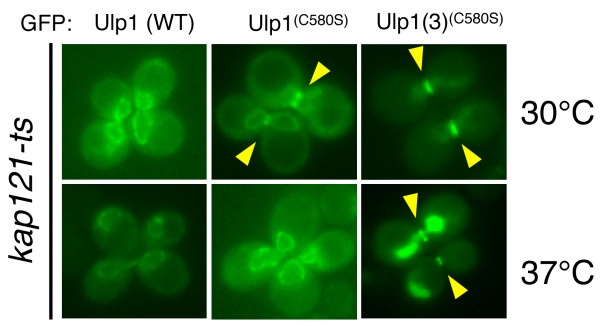
**Kap121-independent small ubiquitin-like modifier (SUMO)-targeting information resides in region 3 of Ulp1**. *kap121ts *cells were transformed with plasmids expressing GFP-tagged wild-type (WT) Ulp1, Ulp1^(C580S) ^and Ulp1(3)^(C580S) ^under the control of the Ulp1 promoter (YOK 1487, YOK 1488 and YOK 1944). Shown are representative images indicating the localization of GFP-tagged Ulp1 constructs in large-budded cells at 30°C and 37°C, the nonpermissive temperature for *kap121-ts*. Arrowheads indicate the position of septin ring-localized Ulp1 constructs.

In the preceding sections, we described our identification of necessary and sufficient substrate-targeting information in the catalytic domain (region 3) of Ulp1. However, region 3 of Ulp1 may not be the only domain involved in targeting to the septin ring. Region 1 of Ulp1, the Kap121-binding domain, has previously been implicated in septin targeting. Specifically, it has been reported that Kap121 is required for targeting Ulp1 to the septin ring during mitosis [27]. Therefore, we decided to assess the role of Kap121 in the substrate-targeting of Ulp1(3)^(C580S)^. Specifically, we used a *kap121ts *mutant [48] to assess the septin ring-targeting of WT Ulp1, full-length Ulp1^C580S ^and Ulp1(3)^(C580S)^. In our analysis, we found that full-length Ulp1^C580S ^required Kap121 function for targeting to the septin ring. At the permissive temperature (30°C), Ulp1^C580S ^demarcated the NE and septin ring of G_2_/M-arrested cells. After a shift to the nonpermissive temperature, however, Ulp1^C580S ^could no longer be detected at the septin ring (Figure 4, middle). Surprisingly, the Ulp1(3)^(C580S) ^truncation was localized to the septin ring at the permissive and nonpermissive temperatures in a *kap121ts *strain. As shown herein, Ulp1(3)^(C580S) ^resided both inside the nucleus and at the septin ring at 30°C and 37°C (Figure 4, right).

Our data suggest that Ulp1 contains both Kap121-dependent and Kap121-independent septin ring-targeting information. The only requirement to detect full-length Ulp1 at the septin ring is the C580S mutation and functional Kap121 (Figures 1, 2 and 4). In contrast Ulp1(3)^(C580S)^, which lacks all domains required for NPC interaction through Kap121, Kap60 and Kap95, localizes to the septin ring and inside the nucleus. This finding provides strong evidence that Kap121-independent septin ring-targeting information resides in the catalytic domain (region 3) of Ulp1.

### Multiple features in the catalytic domain of Ulp1 affect SUMO interactions

**Figure 5:**
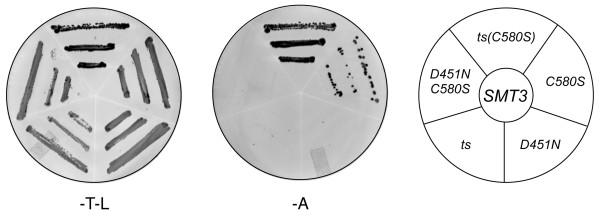
**Distinct and separate features of Ulp1 are required for interaction with SUMO**. Two-hybrid analysis of various Ulp1(3) truncation mutants (C580S: catalytically inactive; D451N: deleted salt bridge with small ubiquitin-like modifier (SUMO); ts: mutations including S450N in *ulp1ts-333*) with SUMO/Smt3-BD. The presence of both Smt3 (pOBD2/TRP1) and Ulp1 constructs (pOAD/LEU2) was confirmed by growth on growth media lacking tryptophan and leucine (-T-L). The interaction between Ulp1 constructs and Smt3 is shown as triplicate patches of cells on media lacking adenine (-A). See Figure 3A for a graphic representation of individual constructs.

Our finding that a single amino acid change in the catalytic domain of Ulp1 results in greatly enhanced, SUMO-dependent localization to the septin ring also prompted us to investigate the two-hybrid interactions of Ulp1 with budding yeast SUMO (Smt3-BD; Smt3 fused to the Gal4 DNA-binding domain). Full-length WT Ulp1, the full-length catalytically inactive Ulp1^C580S ^mutant, the Ulp1 Kap121-interacting domain (region 1), the Ulp1 Kap60/Kap95-interacting domain (region 2) and the catalytic domain (region 3) failed to interact with Smt3-BD (data not shown). However, the catalytically inactive Ulp1(3)^(C580S) ^truncation interacted reproducibly and above background with Smt3 (see Figure 5, C580S).

Ulp1(3)^(C580S) ^appears to interact only weakly with our Smt3 two-hybrid bait construct, as indicated by fewer colonies on the reporter media (Figure 5, Ade). However, such an interpretation assumes an equally available pool of both bait and prey. One possible explanation for this result is that the Ulp1(3)^(C580S) ^two-hybrid prey construct interacts with a number of available substrates in the cell (for example, free Smt3 and other sumoylated proteins), and, as a result, this sequestration is no longer available to the Smt3 two-hybrid bait construct, thus creating the appearance of a weak interaction. We reasoned that introduction of the *ulp1ts *mutations could weaken the potential substrate-trapping phenotype of Ulp1(3)^(C580S)^, making more of the pool available to engage the Smt3 bait construct. Consistent with this model, when we introduced the *ulp1ts *mutations into the substrate-trapping Ulp1(3)^(C580S) ^prey construct, we observed a more robust two-hybrid interaction with the Smt3 bait (Figure 5, compare C580S and ts(C580S). It must also be noted, however, that currently we cannot fully explain the variations in our *in vivo *and *in vitro *assays used to assess ability of Ulp1(3)^(C580S) ^to interact with Smt3.

Next we focused on the D451N mutant of Ulp1 that prevents the interaction of Ulp1 with SUMO [31,47]. As shown above, D451N, when introduced into Ulp1^(C580S)^, prevents localization of this construct to the septin ring (Figure 3A, C580S/D451N). Correspondingly, we found that introduction of the D451N mutation into Ulp1(3)^(C580S) ^completely abolished the two-hybrid interaction with Smt3 (Figure 5, compare C580S, D451N/C580S and D451N). These observations provide evidence that the targeting of Ulp1 to sumoylated substrates is a closely balanced act involving both Smt3 targeting and retention.

### Ulp1(3)^(C580S) ^truncation binds SUMO and SUMO-modified proteins

We hypothesized that if Ulp1(3)^(C580S) ^were to interact avidly with Smt3, this mutated moiety of Ulp1 could efficiently interact with SUMO adducts *in vitro*. Therefore, to test the direct interaction of Ulp1(3)^(C580S) ^with SUMO, we fused this domain to the carboxy terminus of maltose-binding protein (MBP) and expressed the recombinant fusion protein in bacteria. Subsequently, the MBP-Ulp1(3)^(C580S) ^fusion protein was purified from bacterial extracts and bound to amylose resin (see Methods). As a control to assess the ability of MBP-Ulp1(3)^(C580S) ^to interact with sumoylated proteins, we also purified a second MBP-fused Ulp1(3)^(C580S) ^construct lacking the SBS domain (3^(C580S)^ΔSBS).

**Figure 6:**
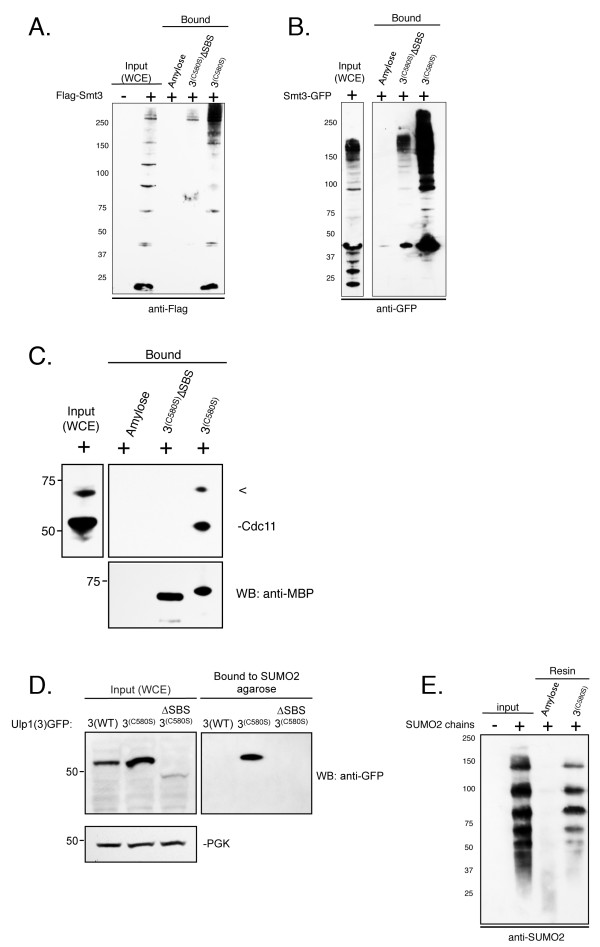
**The Ulp1(3)^(C580S) ^truncation binds SUMO and SUMO-modified proteins**. **(A) **and **(B) **Immobilized Ulp1(3)^(C580S) ^was analyzed for its ability to affinity-purify Smt3 from yeast whole-cell extracts (WCEs). WCEs containing FLAG-tagged Smt3 (YOK 428) (left) or GFP-Smt3 (YOK 1857) (right) (input) were prepared under nondenaturing conditions and incubated with immobilized maltose-binding protein (MBP)-Ulp1(3)^(C580S) ^(3^(C580S)^), MBP-Ulp1(3)^(C580S) ^lacking the small ubiquitin-like modifier (SUMO)-binding surface (3^(C580S)^ΔSBS) or unbound resin (amylose). After washing and elution, bound Smt3 and Smt3 conjugates were detected using either anti-Flag or anti-GFP antibody. **(C) **Immobilized Ulp1(3)^(C580S) ^was analyzed for its ability to affinity-purify Cdc11 from yeast WCEs. WCE containing GFP-Smt3 (YOK 1857) was prepared under nondenaturing conditions and incubated with immobilized MBP-Ulp1(3)^(C580S)^, MBP-Ulp1(3)^(C580S) ^lacking the SUMO-binding surface (3^(C580S)^ΔSBS) or unbound resin (amylose). After washing and dilution, bound Cdc11 was detected using an anti-Cdc11 antibody (Santa Cruz Biotechnology). **(D) **WCEs from logarithmically growing yeast cells expressing GFP-tagged Ulp1(3), Ulp1(3)^(C580S) ^and Ulp1(3)^(C580S)^ΔSBS (YOK 1839, YOK 1907, YOK 1903) (input) were prepared under nondenaturing conditions. Extracts were then incubated with SUMO2 immobilized on agarose beads (Boston Biochem). After washing and elution with sample buffer, bound proteins were detected using an anti-GFP antibody. **(E) **SUMO2 chains (Boston Biochem) were incubated with resin-bound MBP-Ulp1(3)^(C580S) ^or unbound resin (amylose). After washing and elution with sample buffer, bound proteins were detected using an anti-SUMO2 antibody. SUMO2 chains loading control (input). Concentrations of immobilized MBP-Ulp1(3)^(C580S) ^and MBP-Ulp1(3)^(C580S) ^lacking the SUMO-binding surface (3^(C580S)^ΔSBS) were confirmed by Coomassie staining of eluted proteins and quantitation on an Agilent 2100 Bioanalyzer (Agilent Technologies).

First we determined the ability of MBP-Ulp1(3)^(C580S) ^to affinity-purify sumoylated proteins from crude yeast cell extracts. *ulp1ts-333 *cells expressing FLAG-tagged SMT3 were grown to log phase prior to preparation of yeast cell extracts (see Methods). These extracts were then incubated with resin-bound MBP-Ulp1(3)^(C580S)^, MBP-Ulp1(3)^(C580S)^-ΔSBS or unbound amylose resin. After washing, bound yeast proteins were eluted, separated on SDS-PAGE gels and examined by Western blot analysis with an anti-FLAG antibody. Flag-SMT3-modified proteins present in the whole-cell extracts (WCEs) (Figure 6, lane 2) could clearly be detected bound to MBP-Ulp1(3)^(C580S) ^(lane 5) but not to the MBP-Ulp1(3)^(C580S)^-ΔSBS control protein (lane 4). We identified both unconjugated Flag-Smt3 proteins as well as several higher-molecular-weight adducts. These data suggest that Ulp1(3)^(C580S) ^can efficiently bind and enrich sumoylated proteins from crude yeast cell extracts. To demonstrate the versatility of Ulp1(3)^(C580S)^-aided Smt3 purification, we also purified monomeric and conjugated GFP-Smt3 from yeast cells (Figure 6B). Additionally, we probed the extracts and eluted proteins shown in Figure 6B with an anti-Cdc11 antibody, which revealed the specific copurification of Cdc11 with immobilized Ulp1(3)^(C580S) ^(Figure 6C).

In the reciprocal experiment, we tested whether a GFP-tagged Ulp1(3)^(C580S) ^construct expressed in yeast cells could bind immobilized SUMO2, which is highly conserved to yeast Smt3. In this experiment, yeast cells expressing CEN-plasmid levels of GFP-tagged Ulp1(3), Ulp1(3)^(C580S) ^or the Ulp1(3)^(C580S)^-ΔSBS (see Figure 3) were grown to log phase prior to preparation of yeast cell extracts. Individual extracts were then incubated with SUMO2 immobilized on agarose beads (see Methods). After washing, bound yeast proteins were eluted, separated on SDS-PAGE gels and examined by Western blot analysis with an anti-GFP antibody. This time the GFP-tagged Ulp1(3)^(C580S) ^could be detected in the WCEs and bound to the SUMO2 agarose (Figure 6D). In contrast, neither the WT catalytic domain of Ulp1 (Ulp1(3)) nor Ulp1(3)^(C580S)^(SBSΔ) was bound to SUMO2 agarose. Similarly, the Ulp1(3)^(C580S) ^could also be purified on SUMO1 agarose (data not shown).

We also tested whether immobilized Ulp1(3)^(C580S) ^could be used to purify SUMO chains. In this experiment, we incubated purified SUMO2 chains with our immobilized Ulp1(3)^(C580S) ^or the unbound amylose resin. After washing, bound SUMO2 chains were eluted, separated on SDS-PAGE gels and examined by Western blot analysis with an anti-SUMO2 antibody. SUMO2 chains could clearly be detected in the input (Figure 6E, lane 2) and bound the MBP-Ulp1(3)^(C580S) ^(lane 4), but not the resin-only control (Figure 6E, lane 3). Both lower- and higher-molecular-weight adducts of SUMO2 were purified with a preference for higher-molecular-weight chains (5-mer, 6-mer or 7-mer). These data suggest that Ulp1(3)^(C580S) ^can efficiently bind and enrich SUMO2 chains *in vitro *and that the MBP fusion of Ulp1(3)^(C580S) ^may also be useful for the purification of sumoylated proteins from mammalian cells.

### A SUMO2-binding platform for substrate ubiquitination

STUbLs such as the yeast Slx5/Slx8 heterodimer and the human RNF4 protein efficiently ubiquitinated proteins modified with SUMO chains [49,50]. These proteins interact with their respective sumoylated ubiquitinated targets through SIMs. STUbL reactions have been reconstituted *in vitro*, but the purification of target proteins modified with SUMO chains has been technically difficult or prohibitively expensive. The ability of Ulp1(3)^(C580S) ^to interact with SUMO may therefore provide a simple way to purify a SUMO chain-modified STUbL target of choice.

**Figure 7:**
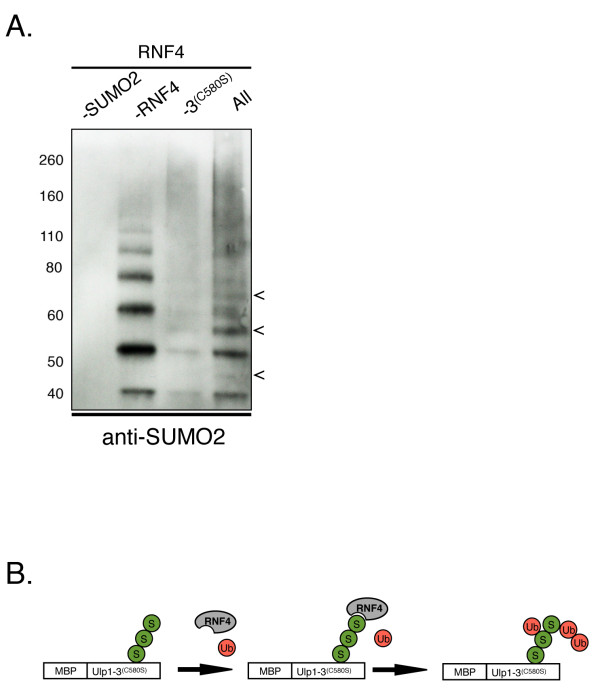
**MBP-Ulp1(3)^(C580S) ^can serve as a SUMO2 binding platform for SUMO-targeted ubiquitin ligase-mediated substrate ubiquitination**. **(A) **SUMO2 chains (Boston Biochem) were incubated with resin-bound maltose-binding protein (MBP)-Ulp1(3)^(C580S)^. The complex of MBP-Ulp1(3)^(C580S) ^with small ubiquitin-like modifier 2 (SUMO2) chains was then eluted and added to an *in vitro *ubiquitination reaction with the SUMO-targeted ubiquitin ligase (STUbL) RNF4, an E3 ubiquitin ligase. Proteins in the STUbL reactions were separated by SDS-PAGE and assessed by Western blot analysis with an anti-SUMO2 antibody. Arrows indicate modified SUMO2 chains. Lane 1: No SUMO chains; lane 2: no RNF4; lane 3: no Ulp1(3)^(C580S)^; lane 4: all reagents. **(B) **Proposed model for using MBP-Ulp1(3)^(C580S) ^as a SUMO2-binding platform for substrate ubiquitination. SUMO2, ubiquitin and RNF4 are indicated by spheres labeled S, spheres labeled Ub and the gray oval labeled RNF4, respectively.

To test whether Ulp1(3)^(C580S) ^can serve as a platform to modify a purified protein with SUMO2 chains, we incubated the immobilized MBP-Ulp1(3)^(C580S) ^with SUMO2 chains. Unbound SUMO2 chains were removed by washing. The MBP-Ulp1(3)^(C580S) ^SUMO2 chain complex was then eluted and added into a STUbL *in vitro *ubiquitinated reaction containing recombinant RNF4 (K A Fryrear and O Kerscher, unpublished reagents). Proteins in the STUbL-mediated ubiquitination assay were separated on SDS-PAGE gels and examined by Western blot analysis with an anti-SUMO2 antibody (Figure 7A). Consistent with previous observations, we were able to detect ubiquitinated SUMO2 chains after the STUbL reaction. This ubiquitination was dependent on RNF4 and SUMO2 chains. On the basis of these results, we propose that Ulp1(3)^(C580S) ^may provide a useful, widely applicable tool for the study of sumoylated proteins and STUbL targets (Figure 7B).

## Discussion

In this study, we have demonstrated that region 3 of Ulp1, the catalytic domain, contains critical information for subcellular targeting to sumoylated substrates, including the septin Cdc11. To determine how Ulp1 is targeted to its substrates, we took advantage of a catalytically inactive Ulp1 mutant (C580S) that exhibited partial redistribution from the NE to the septin ring of dividing yeast cells. We found that the relocalization of Ulp1 depended on functional Smt3 and sumoylated proteins at the septin ring of dividing cells.

Importantly, using this novel Ulp1 *in vivo *septin-ring localization assay, we traced the critical targeting information to two features in region 3 of Ulp1, a previously identified SBS (amino acids 418 to 447) and a SUMO-contacting residue (D451) that resides near the carboxy terminus (see structure supplied). D451 of Ulp1 has previously been shown to contact Smt3 through a salt-bridge interaction [31,47]. Indeed, in our analysis, we provide evidence that the salt bridge-disrupting D451N mutation abolishes the SUMO-dependent targeting of Ulp1 to septins and prevents the two-hybrid interaction of the catalytic domain of Ulp1 with Smt3 (Figures 3A, B and 5).

The sole requirements for the enrichment of full-length Ulp1 at the septin ring were the catalytically inactivating C580S mutation in the catalytic domain of Ulp1 and functional Kap121. This finding has important implications for the targeting role played by the amino-terminal karyopherin-binding domains of Ulp1 (discussed below). Additionally, Smt3 processing appears to be required for substrate release. The catalytically inactive Ulp1(3)^(C580S) ^mutant is predominantly localized to the septin ring and nucleus of dividing yeast cells, whereas the catalytically active WT Ulp1(3) shows merely diffuse staining throughout the cell (compare Figure 3B, top). This is not due to differential expression or stability of either GFP fusion, as similar amounts of proteins were detected in cell extracts (see Figure 6D). We propose and show in our biochemical analysis that the C580S mutant may trap Smt3-modified proteins, allowing it to be observed in association with cellular desumoylation substrates. In support of this assessment, combining the D451N with the C580S mutation abolishes all visible bud-neck localization (Figure 3A). Therefore, we propose that Ulp1^(C580S) ^first targets and docks Smt3 through the SBS domain and the salt bridge-forming D451 residue, then traps it in place because of its inability to cleave after the di-glycine motif of Smt3. We can assume that a trapped substrate prevents further catalysis or interactions with other Smt3 molecules, an assessment that is borne by our finding that, despite its septin-targeting and SUMO-binding properties, Ulp1(3)^(C580S) ^interacts only weakly with Smt3 in a two-hybrid assay (Figure 5 and Additional file 4). A better understanding of how the sumoylated substrate is trapped by Ulp1(3)^(C580S) ^may have important implications for the rational design of inhibitors for Ulp1-like SUMO proteases, but may await the elucidation of the cocrystal structure with a trapped substrate.

The interaction of budding yeast Ulp1 with Smt3 relies on multiple hydrophobic and salt-bridge interactions between the catalytic domain (region 3) of Ulp1 and the carboxy-terminal extension of Smt3. By making multiple contacts with Smt3, Ulp1 is particularly well-suited to interact with a wide variety of sumoylated substrates [22,31]. Other SUMO proteases, Ulp2 and several SENP proteins (Senp1, Senp2, Senp6 and Senp7), are believed to interact noncovalently with their sumoylated substrates through dedicated SIMs [14,22]. On the basis of our structure-function analysis of region 3, Ulp1 seems to employ a unique mode of interaction with Smt3 and sumoylated substrates. Ulp1 does not appear to contain *bona fide *canonical SIMs, and neither of the amino-terminal domains of Ulp1 (regions 1 and 2) interact with Smt3 or become enriched at the septin ring [51]. This assessment is also underscored by the arrangement of Smt3 and Ulp1 in the cocrystal structure [31]. The hydrophobic groove of Smt3 that would interact with a SIM-containing protein is turned away from the domains of Ulp1 that interact with Smt3. Interestingly, this may suggest that Ulp1 can be recruited to proteins that are covalently or noncovalently modified with SUMO and SUMO chains.

Our research demonstrates for the first time that noncovalent interactions between Ulp1 and SUMO are important not only for SUMO binding but also for the cytosolic targeting of this SUMO protease to the bud-neck and potentially to sumoylated septins. Septins are not the only cytosolic substrates of Ulp1 and are arguably the most prevalent [35,38]; therefore, they may be readily scored in our septin ring targeting assay (Figures 3A and 3B). We predict that Ulp1 is also targeted to other cytosolic and septin-bound sumoylated substrate proteins, such as the karyogamy protein Kar9 [33]. However, because of the low local concentrations in comparison to sumoylated septins, these proteins may be hard to detect. We propose, however, that sumoylated proteins that accumulate or aggregate in the cytosol of yeast cells may be readily detectable by Ulp1(3)^(C580S)^.

As detailed below, Ulp1(3)^(C580S) ^also provides a useful tool with which to purify these sumoylated proteins (Figure 6E), and such studies are underway (O Kerscher and Z C Elmore, unpublished data). Our findings provide strong evidence that SUMO, at least in the case of sumoylated proteins at the septin ring, is a required signal for the cytoplasmic targeting of Ulp1. Our alignments of the SBS domain and the juxtaposed salt bridge-forming D451 residue reveal that this mode of targeting may also be conserved in other metazoan Ulp1-like SUMO proteases (Additional file 1).

Though we clearly show herein that Ulp1 becomes enriched at the septin ring, we do not yet fully understand how Ulp1 arrives at this subcellular localization. Our findings support the previous observation [27] that Kap121 plays an important role in promoting Ulp1 targeting to the septin ring. Similar to a previously described Ulp1 mutant that lacks the Kap60/Kap95 binding domain (region 2) [27], the septin ring localization of the full-length Ulp1^(C580S) ^protein described herein is dependent on functional Kap121. It is unlikely that the association with Kap121 shuttles Ulp1 to the septins. Rather, in mitosis, Kap121 becomes associated with a transport inhibitory nucleoporin, Nup53, and may thus exclude Ulp1 access to the inner phase of the NPC [52]. This suggests that, in the absence of Kap121-binding, a fraction of Ulp1 is free to associate with sumoylated septins. Our studies confirm that Ulp1 lacking the Kap60/Kap95-binding domain (region 2) is enriched at the NPC and the septin ring (Figure 3A). We extend these observations by showing that the ability to target sumoylated septins resides in the catalytic domain (region 3) of Ulp1. We found that a Ulp1(3)^(C580S) ^mutant, but not WT Ulp1(3), is enriched at the septin ring in the absence of Kap121. These data suggest that both the Ulp1(3)^(C580S) ^mutant and WT Ulp1(3) can interact with sumoylated septins, but, unlike the subtrate-trapping Ulp1(3)^(C580S) ^mutant, the catalytically active WT Ulp1(3) may be quickly released after desumoylation of the target protein, giving it a diffuse appearance in the cell. How Kap121 helps Ulp1-Δ2 to be retained at the septin ring in the absence of the C580S mutation is currently unclear. Kap121 may theoretically promote the interaction with a septin ring-localized protein. However, the localization of Kap121 to septins has not previously been reported.

One intriguing aspect of our study is the results of our analysis of the substrate-trapping Ulp1(3)^(C580S) ^construct. Three lines of evidence reveal the avid interaction of Ulp1(3)^(C580S) ^with SUMO proteins and sumoylated substrates. First, this Ulp1-derived construct shows a pronounced interaction with the bud-neck, which is composed of sumoylated septins *in vivo*. Second, Ulp1(3)^C580S ^interacts with Smt3 in a two-hybrid assay, whereas WT Ulp1(3) does not. Third, the purified recombinant Ulp1(3)^(C580S) ^protein is a potent affinity-tag for the purification of Smt3 conjugates and SUMO-modified proteins. Furthermore, in preliminary BiaCore analyses, we determined the *K*_d _for the interaction between Ulp1(3)^(C580S) ^and SUMO1 to be 12.8 nM, which is about 200 times stronger than the interaction between a SIM and SUMO, as previously reported by Hecker and co-workers [53]. A related study involving the C603S mutant of the human SENP1 protease confirms our assessment of the substrate-trapping feature. Those authors observed relocalization of their SENP1(C603S) mutant *in vivo *to PML nuclear bodies and domains of the HDAC4 protein, suggesting that SUMO-dependent targeting may be a conserved feature of Ulp1-like SUMO proteases [54]. The latter may also provide a useful strategy for the identification of mitotically important desumoylation substrates. Indeed, two-hybrid screens with Ulp1(3)^(C580S) ^in the laboratory have already identified several novel cytosolic desumoylation targets (M Donaher and O Kerscher, unpublished data). We are also exploring the ability of Ulp1(3)^(C580S) ^to act as a SUMO chain-binding tag that can be used to promote the interaction of putative STUbL target proteins with RNF4 and other STUbLs (Figure 7).

How Ulp1 and other SUMO proteases target specific mitotic substrates for desumoylation remains unknown. Our analysis of SUMO-dependent Ulp1 targeting to the septin ring provides important evidence that Ulp1-like SUMO proteases do not passively await their desumoylation substrates, but rather dynamically localize to them in a cell-cycle-specific manner. Future experiments that take advantage of the SUMO-binding properties of the substrate-trapping Ulp1(3)^(C580S) ^construct may prove useful for the identification of clinically relevant targets of conserved Ulp1-like SUMO proteases in yeast and human cells.

## Conclusions

Ulp1 remains one of the most enigmatic SUMO proteases with an essential role in cell-cycle progression. Our work focuses on the fundamental but unresolved question of how Ulp1, an NPC-associated SUMO protease, targets mitotic desumoylation targets in the cytosol. The current study of Ulp1 reveals for the first time that (1) specific determinants of Ulp1's catalytic domain are utilized to target the septins at the bud-neck of dividing cells, (2) Ulp1 requires SUMO and intact sumoylation for correct cytosolic targeting, (3) Ulp1, unlike Ulp2, relies on specific salt bridge-forming residues for substrate targeting, (4) septin-targeting information in the catalytic domain of Ulp1 is independent of Kap121 and (5) the substrate-targeting domain of Ulp1 can be modified to identify and purify sumoylated substrates of Ulp1 in genetic screens and in biochemical analyses.

## Methods

### Yeast strains, media and plasmids

The yeast strains and plasmids used in this study are listed in Table 1. *smt3-331 *expresses a temperature-sensitive Smt3 protein containing a L26S mutation ([45] and S Brill, personal communication). *smt3R11, 15, 19 *expresses a triple-mutant of *Smt3 *rendered unable to form SUMO chains through replacement of lysines 11, 15 and 19 with arginines [13]. *ulp1ts *expresses a temperature-sensitive mutant of Ulp1 that contains three mutations (I435V, N450S and I504T) in region 3, the catalytic domain [17]. Yeast media preparation and manipulation of yeast cells were performed as described previously [55]. Yeast strains were grown at 30°C unless otherwise indicated. DNA fragments containing Ulp1 under the control of its endogenous promoter were amplified from yeast genomic DNA and placed in-frame with a carboxy-terminal GFP-tag in the CEN/LEU2 plasmid pAA3 [56]. Primer pairs used for full-length Ulp1 amplification were OOK2 (ULP1 (-310 to -294)) and OOK3 (ULP1 (+1, 842 to +1, 863)).

**Table 1:** Yeast strains and plasmids

Name	Pertinent genotypes or parent strain	Plasmids	Study/source
MHY500	*Matα his3-Δ200 leu2-3, 112 ura3-52 lys2-801trp1-1* *gal2*		Li and Hochstrasser, 2003 [26]
BY4743	*MATa leu2Δ0 met15Δ0 ura3Δ0*		Open Biosystems (Huntsville, AL, USA)
YOK 1611	MHY500	*ULP1-GFP/LEU2*	This study
YOK 1474	″	*ULP1^(C580S)^-GFP/LEU2*	″
YOK 1490	″	*ULP1(Reg1)-GFP/LEU2*	″
YOK 1861	″	*UlLP1(Reg2)-GFP/LEU2*	″
YOK 1479	″	*ULP1(Δ2)-GFP/LEU2*	″
YOK 2016	″	*ULP1^(D451N C580S)^-GFP/LEU2*	″
YOK 1839	″	*ULP1(Reg3)-GFP/LEU2*	″
YOK 1907	″	*ULP1(Reg3^(C580S)^)-GFP/LEU2*	″
YOK 1903	″	*ULP1((Reg3ΔSBS)-GFP/LEU2*	″
YOK 2203	″	*ULP1(SBS)-GFP/LEU2*	″
YOK 1828	″	*ULP1((Reg3^(ts)^)-GFP/LEU2*	″
YOK 2157	″	*ULP1((Reg3^(ts C580S)^)-GFP/LEU2*	″
YOK 1857	″	*SMT3-GFP/LEU2*	Panse *et al*., 2003 [25]
YOK 44	*smt3-331*		Biggins *et al*., 2001 [45]
YOK 1995	″	*ULP1^(C580S)^-GFP/LEU2*	This study
YOK 847	*ubc9-1*		Betting and Seufert, 1996 [44]
YOK 2065	″	*ULP1^(C580S)^-GFP/URA3*	This study
YOK 2144	″	*SMT3-GFP/URA3*	″
GBY1	*MAT***a ***smt3 R11, 15, 19*::*TRP1*		Bylebyl *et al*., 2003 [13]
YOK 1910	GBY1	*ULP1^(C580S)^-GFP/LEU2*	This study
yDS880	*MAT***a***-inc ade2-101 his3-200 leu2-1*::*GAL-HO-LEU2 lys2-801 RAD53*::*FLAG-HIS3 siz1*::*NAT* *siz2*::*HPH sml1*::*KAN trp1-63 ura3-52 VII-L*::*TRP-HO site-LYS2*		Schwartz *et al*., 2007 [46]
YOK 2067	″	*ULP1^(C580S)^-GFP/URA3*	This study
YOK 2143	″	*SMT3-GFP/URA3*	″
*kap121ts*	*kap121::ura3::HIS3 ura3*-*52 his3*Δ*200 trp1*-*1 leu2*-*3, 112 lys2*-*801*	*pRS314-kap121-34*	Leslie *et al*., 2002 [48]
YOK 1487	*kap121ts*	*ULP1-GFP/LEU2*	This study
YOK 1488	*kap121ts*	*ULP1^(C580S)^-GFP/LEU2*	″
YOK 1944	*kap121ts*	*ULP1(Reg3^(C580S)^)-GFP/LEU2*	″
AH109	*MATa, trp1-901, leu2-3, 112, ura3-52, his3-200, gal4*Δ, *gal80*Δ, *LYS2::GAL1UAS-GAL1TATA-HIS3, GAL2UAS-GAL2TATA-ADE2, URA3::MEL1UASMEL1TATA-lacZ, MEL1*		(cat. no. 630444; Clontech, Mountain View, CA, USA)
YOK 2212	AH109	*ULP1 (Reg3^(D451N C580S)^)-pOAD/LEU2 + SMT3-pOBD/TRP1*	This study
YOK 2173	″	*ULP1 (Reg3^(C580S)^)-pOAD/LEU2 + SMT3-pOBD/TRP1*	″
YOK 2175	″	*ULP1 (Reg3^(D451N)^)-pOAD/LEU2 + SMT3-pOBD/TRP1*	″
YOK 2177	″	*ULP1 (Reg3^(ts)^)-pOAD/LEU2 + SMT3-pOBD/TRP1*	″
YOK 2181	″	*ULP1 (Reg3^(ts C580S)^)-pOAD/LEU2 + SMT3-pOBD/TRP1*	″
YOK 2183	″	*SMT3-pOAD/LEU2 + SLX5 pOBD/TRP1*	″
YOK 2185	″	*vector-pOAD/LEU2 + vector pOBD/TRP1*	″
YOK 428	*ulp1::KAN (segregant of heterozygous diploid ULP1/ulp1::KAN in BY4743 - (Open Biosystems, Huntsville, AL, USA (cat. no*. YSC1021-671376)	*ulp1ts/TRP/NAT* *GPD-FLAG-SMT3gg/pRS425*	″

To prepare the truncated and mutated Ulp1-GFP constructs listed in Table 1, the QuikChange XL Site-Directed Mutagenesis Kit (Stratagene/Agilent Technologies, La Jolla, CA, USA) and the Phusion Site-Directed Mutagenesis Kit (Finnzymes Oy/Thermo Fisher Scientific, Vantaa, Finland) were used according to the manufacturers' instructions. Primer sequence information for the construction of individual mutants and truncations are available upon request. All constructs were sequenced, verified and/or confirmed by performing complementation assays (for example, see Additional file 3). Additionally, we confirmed that septin rings are intact in strains expressing plasmid-borne Ulp1(3)^C580^, WT Ulp1 and Ulp1(3) (see Additional file 5). For two-hybrid constructs, ORFs of the indicated genes were PCR-amplified and recombined into gapped pOAD and pOBD2 vectors (Yeast Resource Center, Department of Biochemistry and Department of Genome Sciences, University of Washington, Seattle, WA, USA).

To overexpress and purify Ulp1 truncations from bacteria, the respective Ulp1 fragments were PCR-amplified and cloned into pMALc-HT (a gift from Sean T Prigge, Department of Molecular Microbiology and Immunology, The Johns Hopkins University School of Public Health, Baltimore, MD, USA), thereby adding an in-frame MBP module followed by a TEV protease cleavage site and a His^6 ^epitope tag. Ulp1 derivatives were expressed as MBP fusions in BL21 Star (DE3) cells (Invitrogen) containing a pRIL plasmid. The YCp-111 Cdc3-CFP/LEU plasmid was a kind gift from Ryuichi Nishihama of John Pringle's laboratory (Stanford University School of Medicine, Stanford, CA, USA).

### Yeast two-hybrid assays

Gal4-activation domain (AD) fusions of *ULP1 *and the indicated *ULP1 *mutants in pOAD were transformed into the AH109 reporter strain expressing a Gal4 DNA-binding domain (BD) fusion of SMT3 in pOBD. Two-hybrid interactions of serially diluted cells were scored in duplicate on dropout media lacking adenine.

### Pull-down assays, affinity purification and protein extracts

Frozen bacterial cell pellets from 200 ml of isopropyl β-D-1-thiogalactopyranoside-induced BL21 Star (DE3) cells were thawed on ice and resuspended in 2 ml of 1 × PBS containing 1 × Halt Protease Inhibitor Cocktail (catalog no. 78430; Pierce Biotechnology/Thermo Fisher Scientific, Rockford, IL, USA). Ice-cold cells were sonicated using a Branson Sonifier ultrasonic cell disruptor (Branson Ultrasonics, Danbury, CT, USA), and extracts were cleared by centrifugation at 15, 000 rpm for eight minutes at 4°C. Cleared bacterial extracts were added to 15-ml conical tubes and diluted using 4 ml of 1 × PBS containing the protease inhibitor cocktail. MBP-tagged proteins (MBP-Ulp1(3), MBP-Ulp1(3)^(C580S) ^or MBP-Ulp1(3)^(C580S)^ΔSBS) were bound to 5-ml columns containing 300 μl of amylose resin (New England Biolabs, Ipswich, MA, USA) and washed extensively with 1 × PBS. Yeast cell protein extracts containing the indicated target proteins were passed over the amylose resin, and proteins bound to MBP-Ulp1(3), MBP-Ulp1(3)^(C580S) ^or MBP-Ulp1(3)^(C580S)^ΔSBS were eluted with 100 mM maltose or SDS-PAGE sample buffer. Yeast cell protein extracts were generated by bead-beating about 50 nm of yeast cell pellets in 1 × cell lysis buffer (catalog no. 9803; Cell Signaling Technology, Danvers, MA, USA) containing 25 mM *N*-ethylmaleimide. "Bound" material (Figures 6A and 6B) corresponds to proteins affinity-purified from approximately 8 nm of extracted cells. Input (WCE) lysates correspond to 1.25 nm of extracted proteins (about one-sixth the material loaded onto the column). In SUMO pull-down experiments, recombinant MBP-Ulp1(3)^(C580S) ^or MBP-Ulp1(3) was incubated with SUMO1 or SUMO2 agarose (Boston Biochem Inc, Cambridge, MA, USA) in 1 ml of 1 × PBS with protease inhibitors. Proteins bound to the agarose beads were washed in 1 × PBS and eluted with 1 × SDS-PAGE sample buffer. All protein extracts were separated on NuPage Novex 4-12% Bis-Tris gradient gels (NP0321; Invitrogen, Carlsbad, CA, USA) using 3-morpholinopropane-1-sulfonic acid-SDS running buffer (NP0001; Invitrogen). Equal loading and concentrations of recombinant proteins were confirmed by Coomassie staining and analysis using an Agilent 2100 Bioanalyzer using a Protein 230 Kit (Agilent Technologies, Santa Clara, CA, USA). BiaCore measurements were conducted by Affina Biotechnologies, Inc (Stamford, CT, USA) as a paid service.

### Fluorescence microscopy

Unless otherwise noted, cells were grown in rich media, arrested in G_2_/M using nocodazole (15 μg/ml/3 hours/30°C), washed in 2% dextrose and harvested by centrifugation. Images of live cells were collected using a Zeiss Axioskop microscope (Carl Zeiss USA, Thornwood, NY, USA) fitted with a QImaging Retiga™ SRV charge-coupled device digital camera (QImaging, Surrey, BC, Canada), i-Vision-Mac software (BioVision Technologies, Exton, PA, USA) and a Uniblitz electro-programmable shutter assembly system (Vincent Associates/UNIBLITZ, Rochester, NY, USA). Pertinent filter sets for the above applications include CZ909 (GFP), XF114-2 (CFP) and XF104-2 (YFP) (Chroma Technology Group, Bellows Falls, VT, USA). Images were normalized using i-Vision-Mac software and pseudocolored and adjusted using Adobe Photoshop software (Adobe Systems Inc, San Jose, CA, USA).

### *In vitro *ubiquitination reactions, recombinant proteins and antibodies

Enzymes and substrates used in our *in vitro *ubiquitination assays were quantified using a Protein 230 Kit on the Agilent 2100 Bioanalyzer (Agilent Technologies) according to the manufacturer's instructions. We used 10 × ubiquitination buffer, E1 enzyme (Uba1), ATP and 20 × ubiquitin provided in a commercial ubiquitin activating kit (BML-UW0400-0001; Enzo Life Sciences Inc, Farmingdale, NY, USA). Ubiquitination buffer, isopentenyl diphosphate (100 U/ml), dithiothreitol (DTT) (50 μM), E1 (Uba1), E2 (Ubc4) and E3 enzymes (RNF4) were combined with purified SUMO2 chains (ULC-210; Boston Biochem) and ubiquitin. Reactions totaled 27 μl and were incubated at 30°C for three hours. Reactions were stopped by adding an equal volume of SUMEB with Tris (SUTEB) sample buffer (0.01% bromophenol blue, 10 mM ethylenediaminetetraacetic acid, 1% SDS, 50 mM Tris, pH 6.8, 8 M urea) containing DTT (5 μl of 1 M DTT mixed with 1 ml SUTEB sample buffer). Protein products were boiled in a 65°C heat block for ten minutes and examined by Western blot analysis with anti-human SUMO2 antibody, anti-human SUMO2 (BML-PW0510-0025; Enzo Life Sciences Inc), anti-GFP (JL-8 632380; Clontech, Mountain View, CA), ANTI-FLAG M2 (F3165; Sigma-Aldrich, St Louis, MO, USA), anti-phosphoglycerate kinase (22C5D8; Invitrogen), anti-Cdc11 (y415) (sc-7170; Santa Cruz Biotechnology, Santa Cruz, CA, USA).

## Abbreviations

AD: Gal4-activation domain; BD: Gal4-binding domain; GFP: green fluorescent protein; *K*_d_: dissociation constant; MBP: maltose-binding protein; NPC: nuclear pore complex; ORF: open reading frame; PBS: phosphate-buffered saline; SBS: SUMO binding surface; STUbL: SUMO-targeted ubiquitin ligase; SUMO: small ubiquitin-like modifier; UD: ubiquitin-like protease domain; Ulp1: ubiquitin-like protease 1; YFP: yellow fluorescent protein.

## Authors' contributions

OK and ZE designed and interpreted all the results of the experiments and wrote the manuscript. ZE carried out all experiments with the following exceptions: MD and JW carried out the two-hybrid assays and helped to interpret the results, BM designed and conducted the STUbL assay and HM aligned Ulp SUMO protease sequences, confirmed conserved residues and helped with editing. All authors read and approved the final manuscript.

## Supplementary Material

Additional file 1 **Supplementary Figure S1**. **(A) **Identification of important features required for Ulp1 targeting and small ubiquitin-like modifier (SUMO) binding. The yeast Ulp1 catalytic domain was BLASTed against all nonredundant protein sequences in the National Center for Biotechnology Information database using psi-blast http://blast.ncbi.nlm.nih.gov/Blast.cgi. After seven iterations, the top 100 query sequences (only 11 are shown) were aligned, which included a variety of animal, plant and fungal species. Residues that constitute the SUMO-binding surface (SBS) are shown in red. Also indicated are the salt bridge forming D451 and one of the residues mutated in the *ulp1ts *allele, N450. Conservation: Conservation of amino acid properties. Quality: Alignment quality based on Blosum 62 scores. High values suggest no or conservative mutations. Consensus: Percentage identity. All values were calculated using Jalview http://www.jalview.org/[59]. **(B) **Consensus SBS based on the alignment of 250 sequences from 81 species. The height of the letters corresponds to the frequency of the amino acid in the alignment. The width is based on the proportion of sequences that contain a character (many gaps lead to narrow letters). Also indicated are the salt bridge forming D451 and one of the residues mutated in the *ulp1ts *allele, N450 (WebLogo 3; http://weblogo.threeplusone.com/) [60]. Click here for file

Additional file 2 **Supplementary Figure S2**. Three dimensional representation of the cocrystal structure of the catalytic domain of Ulp1 (Ulp1(3), magenta) with yeast small ubiquitin-like modifier (SUMO) (Smt3, blue). N450, D451 and C580 are indicated in yellow and labeled with the appropriate amino acids. Also shown is the SUMO-binding surface (SBS). Click here for file

Additional file 3 **Supplementary Figure S3**. Complementation analysis of Ulp1-GFP fusion constructs. ULP1-GFP and ULP1(3)-GFP fusion constructs complement the growth phenotype of a *ulp1 *deletion strain. A *ulp1 *shuffle strain (*ulp1::HIS3, ULP1/URA3*) was transformed with one of the following low-copy plasmids: *ULP1-GFP, ULP1-region 1-GFP, ULP1-region 2-GFP, ULP1(3)-GFP, ULP1(3)C580S-GFP *or the empty vector. Transformants were streaked onto yeast extract peptone dextrose medium and then medium containing 5-fluoroorotic acid to select for loss of the *ULP1/URA3 *plasmid. Note that, as expected, only the ULP1-GFP and ULP1(3)-GFP fusion constructs complement the growth phenotype of the *ulp1 *deletion strain. Click here for file

Additional file 4 **Supplementary Figure S4**. Two-hybrid analysis of Ulp1(3)C580S and Ulp1(3)C580A (isolates 1A and 3A) with small ubiquitin-like modifier (SUMO)/Smt3-BD. The presence of both Smt3 (pOBD2/TRP1) and Ulp1 constructs (pOAD/LEU2) was confirmed by growth on growth media lacking tryptophan and leucine (right plate). The interaction between the indicated Ulp1 constructs and Smt3 is shown as triplicate patches of cells on media lacking adenine (left plate). Click here for file

Additional file 5 **Supplementary Figure S5**. Analysis of septin rings in yeast strains expressing various Ulp1 constructs. Strains expressing Ulp1(3)^(C580S)^, full-length Ulp1(WT) or Ulp1(3) were fixed and attached to glass slides. The septin Cdc11 was detected using an anti-Cdc11 antibody. Bud-neck localized Cdc11, 4', 6-diamidino-2-phenylindole-stained nuclear DNA and the overlaid images with pseudocolored red septins and blue DNA are shown. Note that all strains show bud-neck, localized, well-defined septin rings. Click here for file
