# Supplementary figures and images for: Sumo-dependent substrate targeting of the SUMO protease Ulp1
Source: BMC Biol. 2011 Oct 28;9:74. doi: 10.1186/1741-7007-9-74 (PMC3216068; doi:10.1186/1741-7007-9-74)

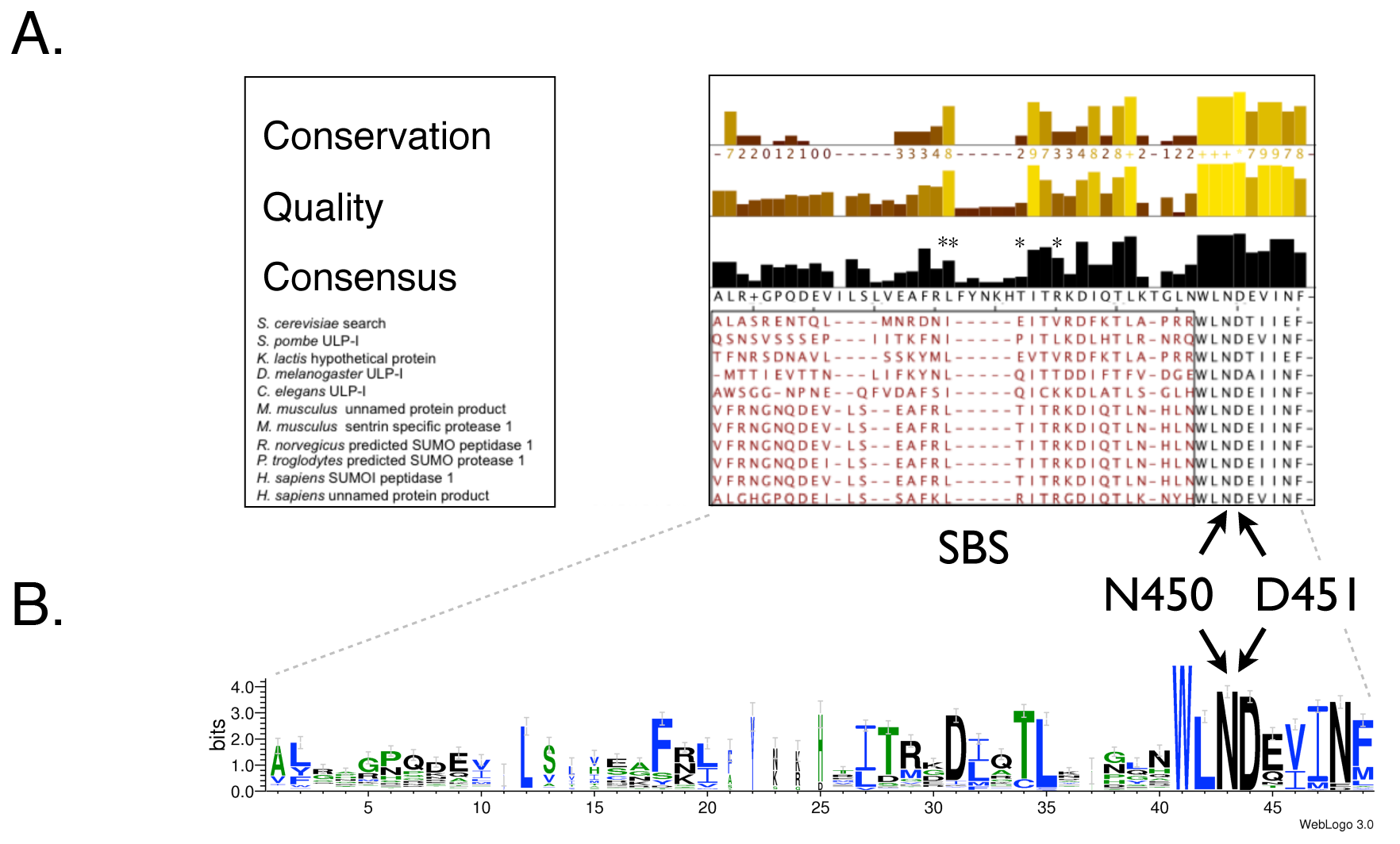

Supplement: Additional file 1 — Supplementary Figure S1. (A) Identification of important features required for Ulp1 targeting and small ubiquitin-like modifier (SUMO) binding. The yeast Ulp1 catalytic domain was BLASTed against all nonredundant protein sequences in the National Center for Biotechnology Information database using psi-blast http://blast.ncbi.nlm.nih.gov/Blast.cgi. After seven iterations, the top 100 query sequences (only 11 are shown) were aligned, which included a variety of animal, plant and fungal species. Residues that constitute the SUMO-binding surface (SBS) are shown in red. Also indicated are the salt bridge forming D451 and one of the residues mutated in the ulp1ts allele, N450. Conservation: Conservation of amino acid properties. Quality: Alignment quality based on Blosum 62 scores. High values suggest no or conservative mutations. Consensus: Percentage identity. All values were calculated using Jalview http://www.jalview.org/[59]. (B) Consensus SBS based on the alignment of 250 sequences from 81 species. The height of the letters corresponds to the frequency of the amino acid in the alignment. The width is based on the proportion of sequences that contain a character (many gaps lead to narrow letters). Also indicated are the salt bridge forming D451 and one of the residues mutated in the ulp1ts allele, N450 (WebLogo 3; http://weblogo.threeplusone.com/) [60]. [file 1741-7007-9-74-S1.TIFF]

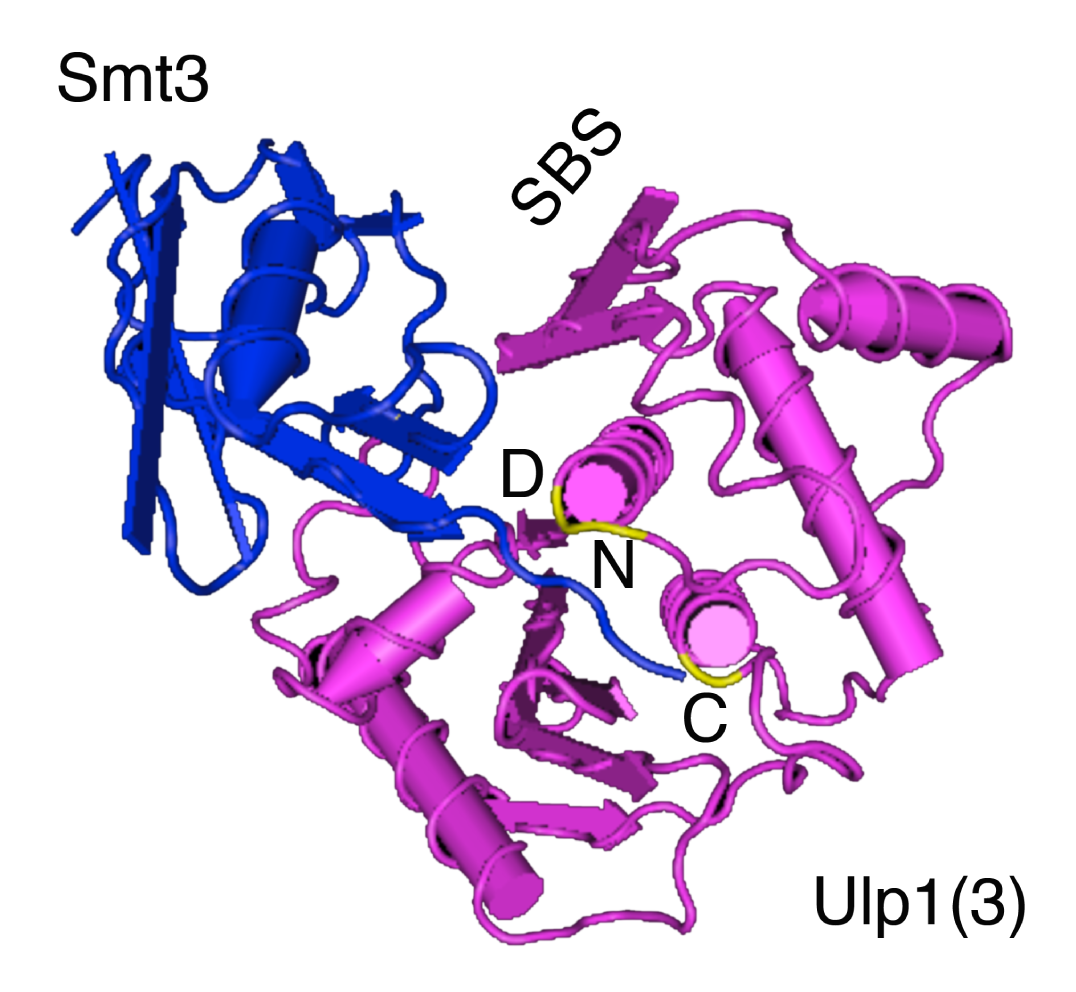

Supplement: Additional file 2 — Supplementary Figure S2. Three dimensional representation of the cocrystal structure of the catalytic domain of Ulp1 (Ulp1(3), magenta) with yeast small ubiquitin-like modifier (SUMO) (Smt3, blue). N450, D451 and C580 are indicated in yellow and labeled with the appropriate amino acids. Also shown is the SUMO-binding surface (SBS). [file 1741-7007-9-74-S2.TIFF]

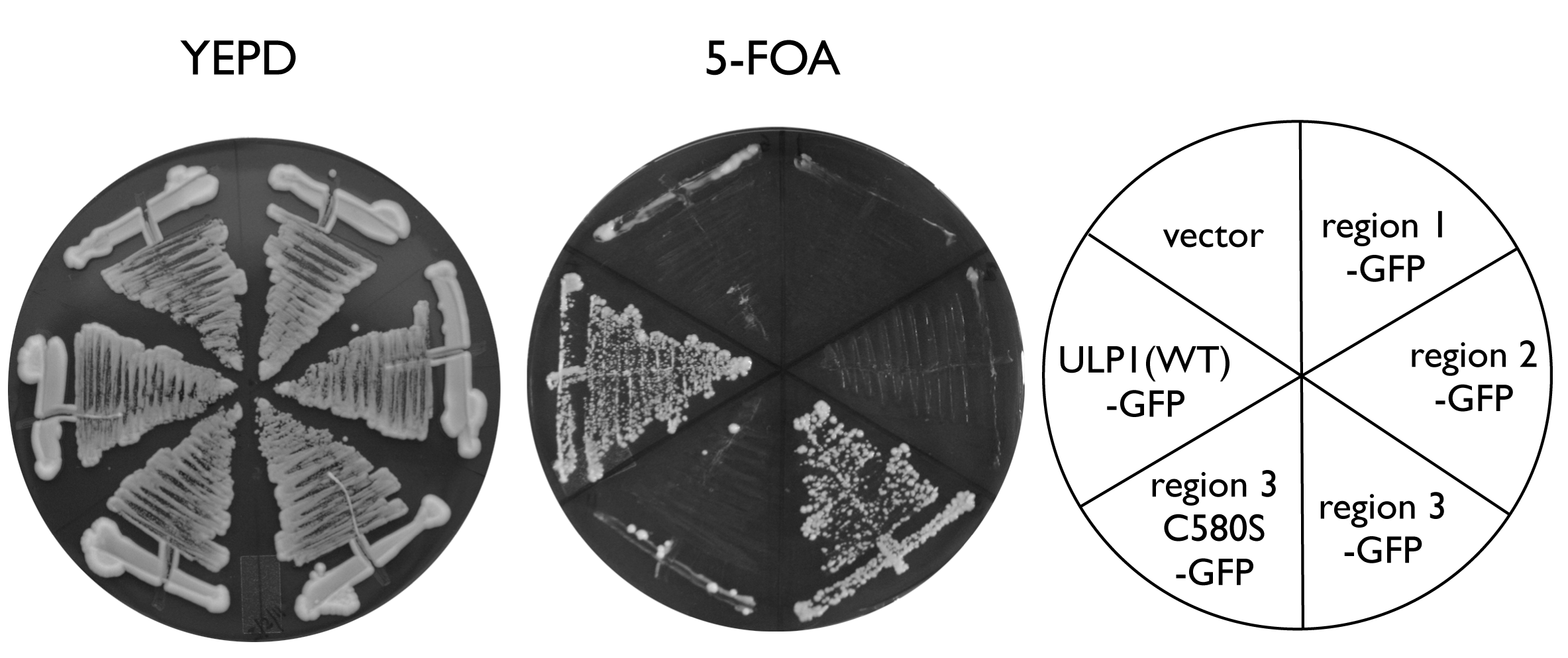

Supplement: Additional file 3 — Supplementary Figure S3. Complementation analysis of Ulp1-GFP fusion constructs. ULP1-GFP and ULP1(3)-GFP fusion constructs complement the growth phenotype of a ulp1 deletion strain. A ulp1 shuffle strain (ulp1::HIS3, ULP1/URA3) was transformed with one of the following low-copy plasmids: ULP1-GFP, ULP1-region 1-GFP, ULP1-region 2-GFP, ULP1(3)-GFP, ULP1(3)C580S-GFP or the empty vector. Transformants were streaked onto yeast extract peptone dextrose medium and then medium containing 5-fluoroorotic acid to select for loss of the ULP1/URA3 plasmid. Note that, as expected, only the ULP1-GFP and ULP1(3)-GFP fusion constructs complement the growth phenotype of the ulp1 deletion strain. [file 1741-7007-9-74-S3.TIFF]

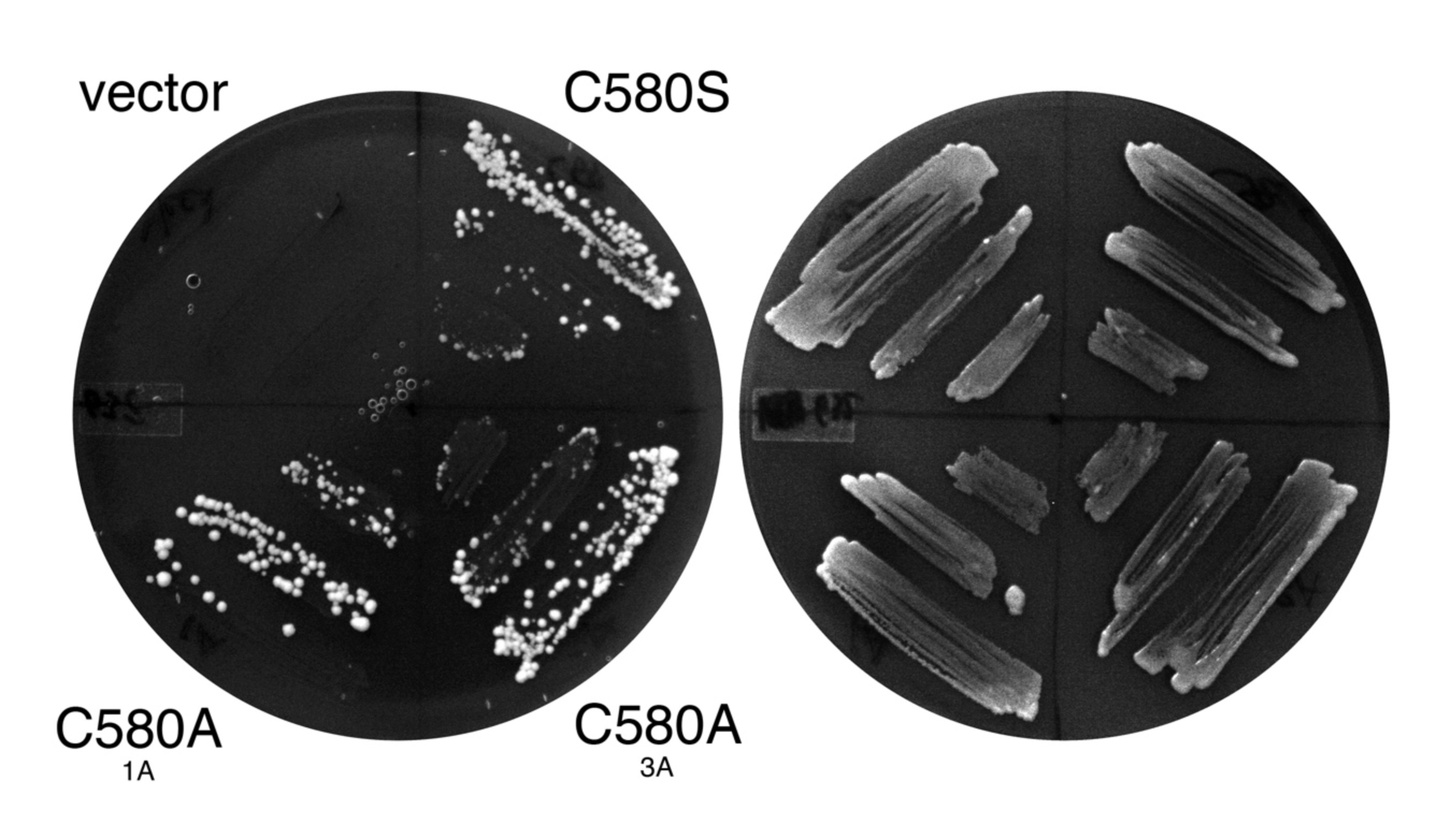

Supplement: Additional file 4 — Supplementary Figure S4. Two-hybrid analysis of Ulp1(3)C580S and Ulp1(3)C580A (isolates 1A and 3A) with small ubiquitin-like modifier (SUMO)/Smt3-BD. The presence of both Smt3 (pOBD2/TRP1) and Ulp1 constructs (pOAD/LEU2) was confirmed by growth on growth media lacking tryptophan and leucine (right plate). The interaction between the indicated Ulp1 constructs and Smt3 is shown as triplicate patches of cells on media lacking adenine (left plate). [file 1741-7007-9-74-S4.TIFF]

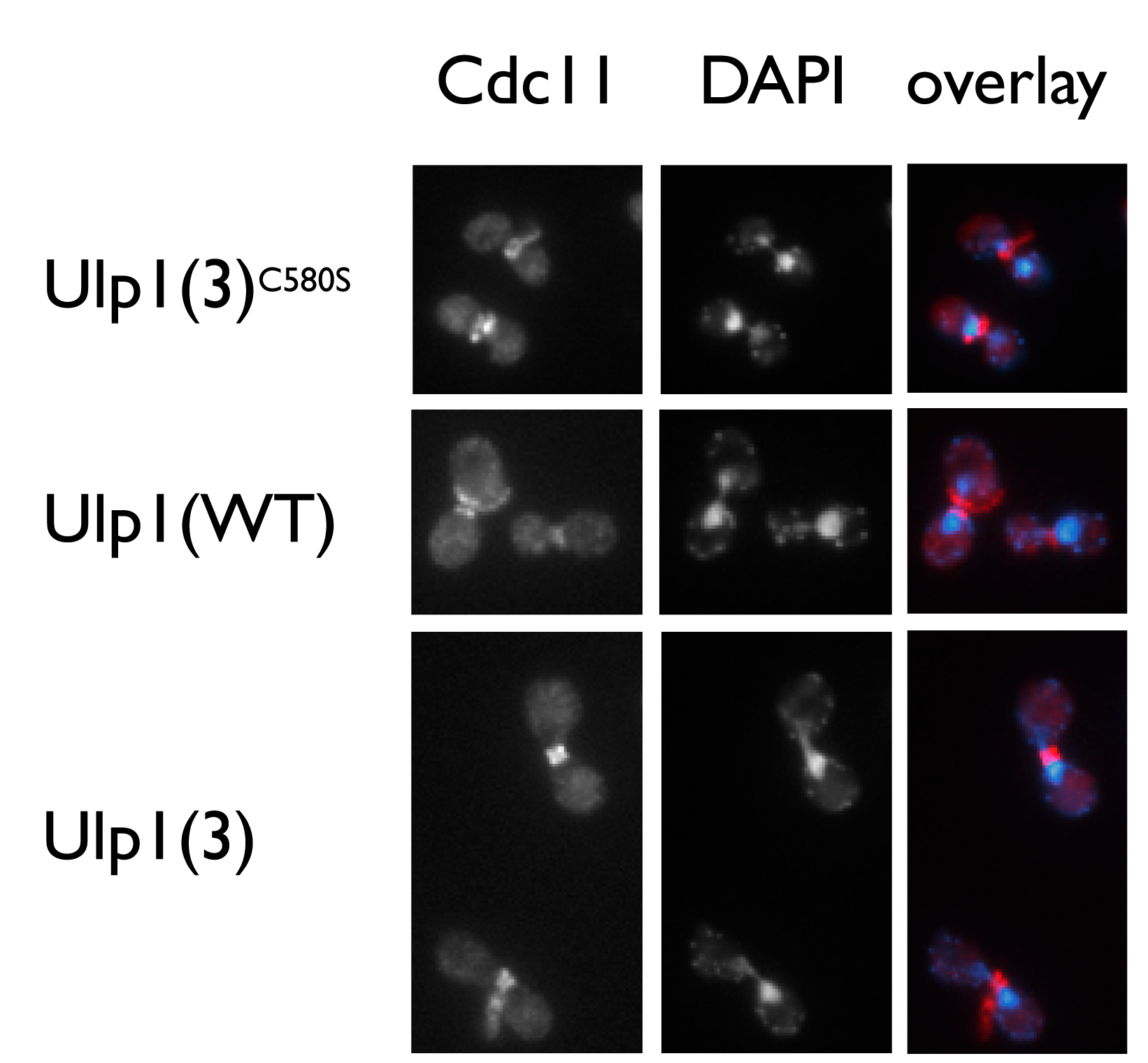

Supplement: Additional file 5 — Supplementary Figure S5. Analysis of septin rings in yeast strains expressing various Ulp1 constructs. Strains expressing Ulp1(3)(C580S), full-length Ulp1(WT) or Ulp1(3) were fixed and attached to glass slides. The septin Cdc11 was detected using an anti-Cdc11 antibody. Bud-neck localized Cdc11, 4', 6-diamidino-2-phenylindole-stained nuclear DNA and the overlaid images with pseudocolored red septins and blue DNA are shown. Note that all strains show bud-neck, localized, well-defined septin rings. [file 1741-7007-9-74-S5.TIFF]
